# Protein Tyrosine Phosphatase 1B Deficiency Potentiates PERK/eIF2α Signaling in Brown Adipocytes

**DOI:** 10.1371/journal.pone.0034412

**Published:** 2012-04-03

**Authors:** Ahmed Bettaieb, Kosuke Matsuo, Izumi Matsuo, Shuo Wang, Ramzi Melhem, Antonis E. Koromilas, Fawaz G. Haj

**Affiliations:** 1 Department of Nutrition, University of California Davis, Davis, California, United States of America; 2 Lady Davis Institute for Medical Research, Sir Mortimer Davis-Jewish General Hospital, Montreal, Quebec, Canada; 3 Department of Oncology, Faculty of Medicine, McGill University, Montreal, Quebec, Canada; Wayne State University, United States of America

## Abstract

**Background:**

Protein-tyrosine phosphatase 1B (PTP1B) is a physiological regulator of glucose homeostasis and body mass, and has been implicated in endoplasmic reticulum (ER) stress. Herein, we assess the role of PTP1B in ER stress in brown adipocytes, which are key regulators of thermogenesis and metabolic response.

**Methodology/Principal Findings:**

To determine the role of PTP1B in ER stress, we utilized brown adipose tissue (BAT) from mice with adipose-specific PTP1B deletion, and brown adipocytes deficient in PTP1B and reconstituted with PTP1B wild type (WT) or the substrate-trapping PTP1B D181A (D/A) mutant. PTP1B deficiency led to upregulation of PERK-eIF2α phosphorylation and IRE1α-XBP1 sub-arms of the unfolded protein response. In addition, PTP1B deficiency sensitized differentiated brown adipocytes to chemical-induced ER stress. Moreover, PERK activation and tyrosine phosphorylation were increased in BAT and adipocytes lacking PTP1B. Increased PERK activity resulted in the induction of eIF2α phosphorylation at Ser51 and better translatability of ATF4 mRNA in response to ER stress. At the molecular level, we demonstrate direct interaction between PTP1B and PERK and identify PERK Tyr615 as a mediator of this association.

**Conclusions:**

Collectively, the data demonstrate that PTP1B is a physiologically-relevant modulator of ER stress in brown adipocytes and that PTP1B deficiency modulates PERK-eIF2α phosphorylation and protein synthesis.

## Introduction

Metabolic syndrome, cardiovascular disease and type 2 diabetes are complex disorders that are associated with obesity and sedentary life style [1], [2]. The mechanisms by which excess nutrients and adiposity trigger changes that lead to these chronic diseases are still being elucidated. Endoplasmic reticulum (ER) is an organelle that is highly responsive to nutrient and energy status of the cell and plays an important role in folding and maturation of newly synthesized proteins. ER dysfunction is a major contributor to metabolic disease [3], [4]. When the folding capacity of the ER is exceeded, misfolded/unfolded proteins accumulate and lead to ER stress [5]. Notably, prolonged high fat-feeding and genetic obesity in mice lead to increased ER stress in liver and adipose tissue [3]. Importantly, obese individuals exhibit increased expression of multiple markers of ER stress in adipose tissue [6]. However, it is not presently clear whether ER dysfunction is a cause or an effect of obesity. Thus, understanding the function of ER stress in insulin resistance and obesity may be of therapeutic potential for the treatment of metabolic disorders.

Cells use adaptive mechanisms to counter the deleterious effects of ER stress known as the unfolded protein response (UPR) [7]. UPR consists of three branches that are controlled by the ER transmemberane proteins PKR-like ER-regulated kinase (PERK), inositol requiring protein 1α (IRE1α), and activating transcription factor 6 (ATF6) [4], [8], [9]. These sensor proteins respond to changes in protein-folding status and activate distinct and sometimes overlapping pathways. PERK phosphorylates the α-subunit of translation initiation factor 2 (eIF2α) at serine 51, a modification that blocks initiation of mRNA translation in response to ER stress [10]–[12]. PERK is a serine/threonine kinase whose autophosphorylation at threonine (Thr980) within its activation loop is essential for its activation and eIF2α phosphorylation [11]. However, PERK is a tyrosine phosphorylated protein [13] and its activity is regulated by tyrosine phosphorylation in as much as autophosphorylation of PERK at tyrosine 615 is required for maximal kinase activity *in vitro* and *in vivo* [14]. IRE1α activation leads to the unconventional splicing of X-box binding protein 1 (XBP1) mRNA leading to the synthesis of a nuclear XBP1 form which induces the transcription of genes encoding ER chaperones [15]–[17]. The third canonical branch of ER stress signaling includes the ATF6 transcription factors [18], [19]. These UPR arms synergize to attenuate stress by increasing the folding capacity of the ER through mechanisms that involve translational attenuation, regulation of ER biogenesis as well as ER-associated protein degradation [5]. However, if the compensatory mechanisms fail to restore homeostasis, ER stress-insuced apoptosis commences [20], [21].

Protein-tyrosine phosphatase 1B (PTP1B) is a widely expressed non-receptor tyrosine-specific phosphatase that is localized on the cytoplasmic face of the ER [22]–[24]. PTP1B is a physiological regulator of glucose homeostasis and energy balance. Specifically, whole-body PTP1B knockout (KO) mice are hypersensitive to insulin, lean and resistant to high fat diet (HFD)-induced obesity [25], [26]. PTP1B has been implicated in the regulation of ER stress signaling. Mouse embryonic fibroblasts lacking PTP1B exhibit impaired IRE1α signaling and attenuated ER stress-induced apoptosis [27]. In addition, liver-specific PTP1B deficiency protects mice against HFD-induced ER stress [28], [29]. On the other hand, PTP1B overexpression in insulinoma MIN6 β-cells mitigates chemical-induced PERK/eIF2α signaling, and PTP1B deficiency increases ER stress-induced cell death [30]. However, the role of PTP1B in regulating ER stress in the adipose tissue remains unexplored.

Adipose tissue integrates an array of homeostatic processes and is a regulator of systemic insulin sensitivity and energy metabolism [31]. White adipose tissue (WAT) is the primary site for triglyceride storage or fatty acid release in response to energy requirements whereas brown adipose tissue (BAT) generates heat via mitochondrial uncoupling of lipid oxidation [32]. BAT is a thermogenic tissue with an established role in the defense against cold, a process known as non shivering thermogenesis [33]. In addition, BAT is recognized for its anti-obesity properties given that mice with increased BAT gain less weight, are more insulin sensitive, and are protected from diabetes [34]–[37]. Interest in BAT has gained traction from recent findings that humans have BAT depots and that their activity varies depending on adiposity, temperature, and age [38]–[41].

In this study, we investigated the role of PTP1B in ER stress signaling in brown adipocytes. We utilized BAT and brown preadipocyte cell lines lacking PTP1B to investigate its role in regulating UPR and in particular PERK-eIF2α phosphorylation in response to ER stress.

## Results

### PTP1B deficiency enhanced UPR in BAT and in differentiated brown adipocytes

Obese rodents and humans exhibit increased ER stress in adipose tissue [3], [6]. Understanding the role of adipose tissue ER stress in metabolic regulation may be of therapeutic potential. BAT is a thermogenic tissue that is recognized for its anti-obesity properties [34]–[36]. We recently demonstrated that PTP1B is a modulator of brown fat adipogenesis through a PPARγ-dependent mechanism and that brown adipocyte differentiation requires regulated expression of PTP1B [42].

**Figure 1:**
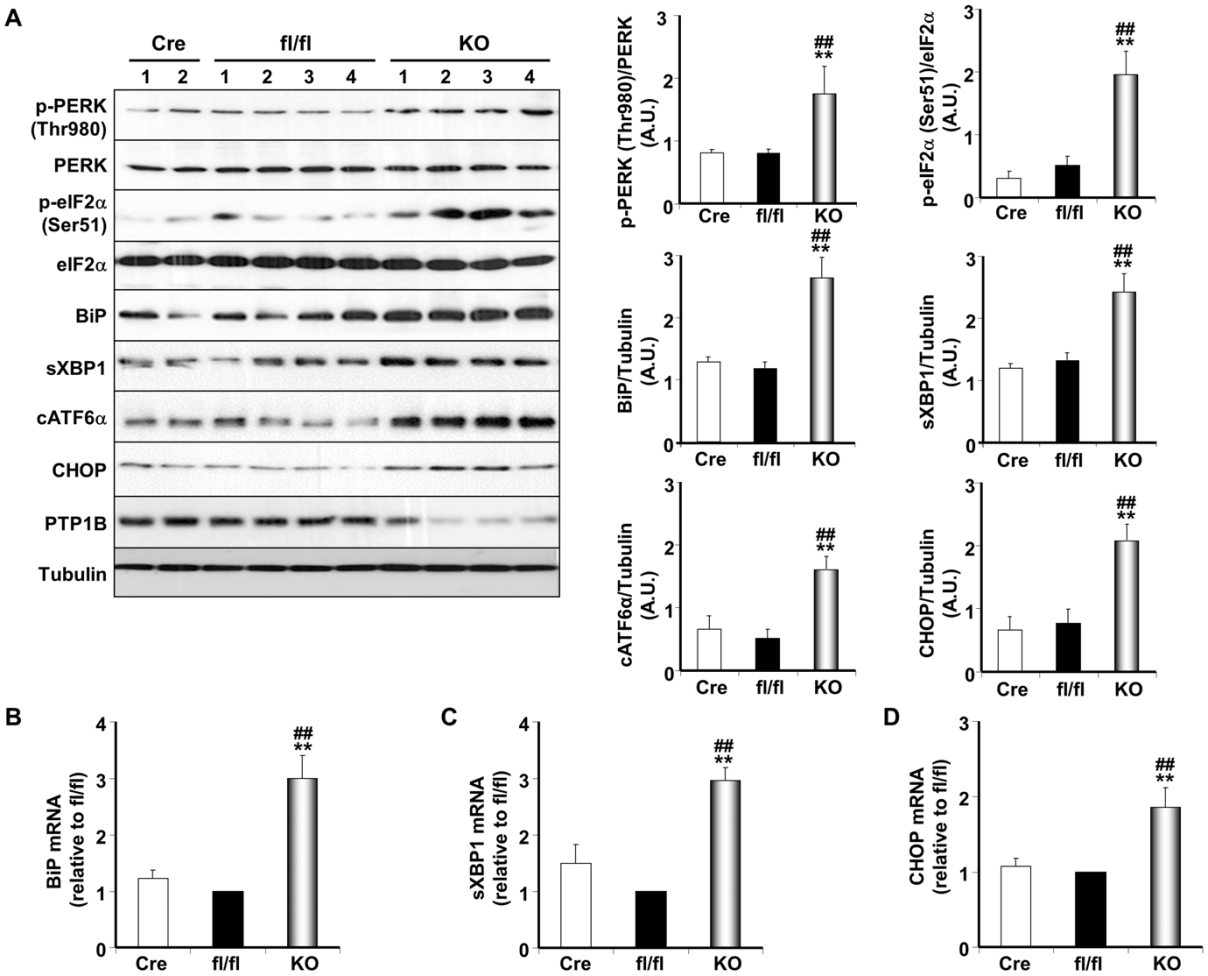
Enhanced UPR in PTP1B-deficient BAT. (**A**) Immunoblots of p-PERK (Thr980), PERK, p-eIF2α (Ser51), eIF2α, BiP, sXBP1, cATF6α and CHOP in BAT lysates of Adipoq-Cre, fl/fl and adipose-PTP1B deficient (KO) mice fed HFD (for 26 weeks). Lysates were also probed for PTP1B to evaluate deletion efficiency, and Tubulin to control for loading. Each lane represents brown adipose tissue from a different animal. Bar charts represent p-PERK (Thr980) and p-eIF2α (Ser51) normalized to their protein expression, BiP, sXBP1, cATF6α normalized to Tubulin, from three independent experiments. (**B–D**) BiP, sXBP1 and CHOP mRNA was measured by quantitative real-time PCR and normalized against GAPDH in BAT from the same cohort of mice. Data represent means ± SEM from at least three different mice per genotype. (*) indicates significant difference between KO and fl/fl, and (^#^) indicates significant difference between KO and Cre.

We initially investigated the effects of ER stress induction on PTP1B protein expression in adipose tissue and adipocytes. Mice fed a HFD exhibited significant increase in PTP1B expression in BAT and WAT depots compared with those fed standard chow diet (Fig. S1A). In addition, palmitate treatment of differentiated brown and white adipocytes led to significant increase in PTP1B expression compared with untreated control (Fig. S1B). To address the role of PTP1B in adipose tissue ER stress, we utilized mice with adipose-specific PTP1B deletion. Adipose-deficient PTP1B mice were generated by crossing PTP1B^fl/fl^ (fl/fl) mice to those expressing Cre recombinase under the control of the adiponectin locus (Adipoq-Cre). The resulting Adipoq-Cre PTP1B^fl/+^ mice were crossed to PTP1B^fl/fl^ mice, yielding Adipoq-Cre PTP1B^fl/fl^ (hereafter termed KO). Control (Cre and fl/fl) and KO mice were maintained on HFD for 26 weeks. KO mice exhibited significant (∼80%) PTP1B deletion in BAT; the residual PTP1B expression in KO BAT likely reflects expression in other cell types in adipose tissue, such as vascular endothelial cells and macrophages (Fig. 1A).

Induction of UPR in BAT by high fat feeding was assessed by immunoblot analysis of specific markers of ER stress. PTP1B deficient BAT exhibited increased PERK activity as indicated by enhanced PERK autophosphorylation at Thr980 as well as elevated phosphorylation of its substrate eIF2α at Ser51 compared with control mice (Fig. 1A). Expression of the molecular chaperone BiP was also increased in PTP1B deficient BAT compared with control. In addition, PTP1B deficiency resulted in increased expression of spliced XBP1, cleaved ATF6α and CHOP proteins (Fig. 1A). Moreover, BiP, sXBP1 and CHOP mRNAs were also upregulated in PTP1B deficient BAT compared with control (Fig. 1B–D). Consistent with findings in BAT, subcutaneous WAT depot of KO mice exhibited increased PERK Thr980 and eIF2α Ser51phosphorylation and increased BiP, sXBP1, cATF6α and CHOP expression compared with control (Fig. S2A). In addition, retroperitoneal WAT depot of KO mice exhibited increased PERK Thr980 and eIF2α Ser51 phosphorylation and increased cATF6α and CHOP expression compared with control (Fig. S2B).

**Figure 2:**
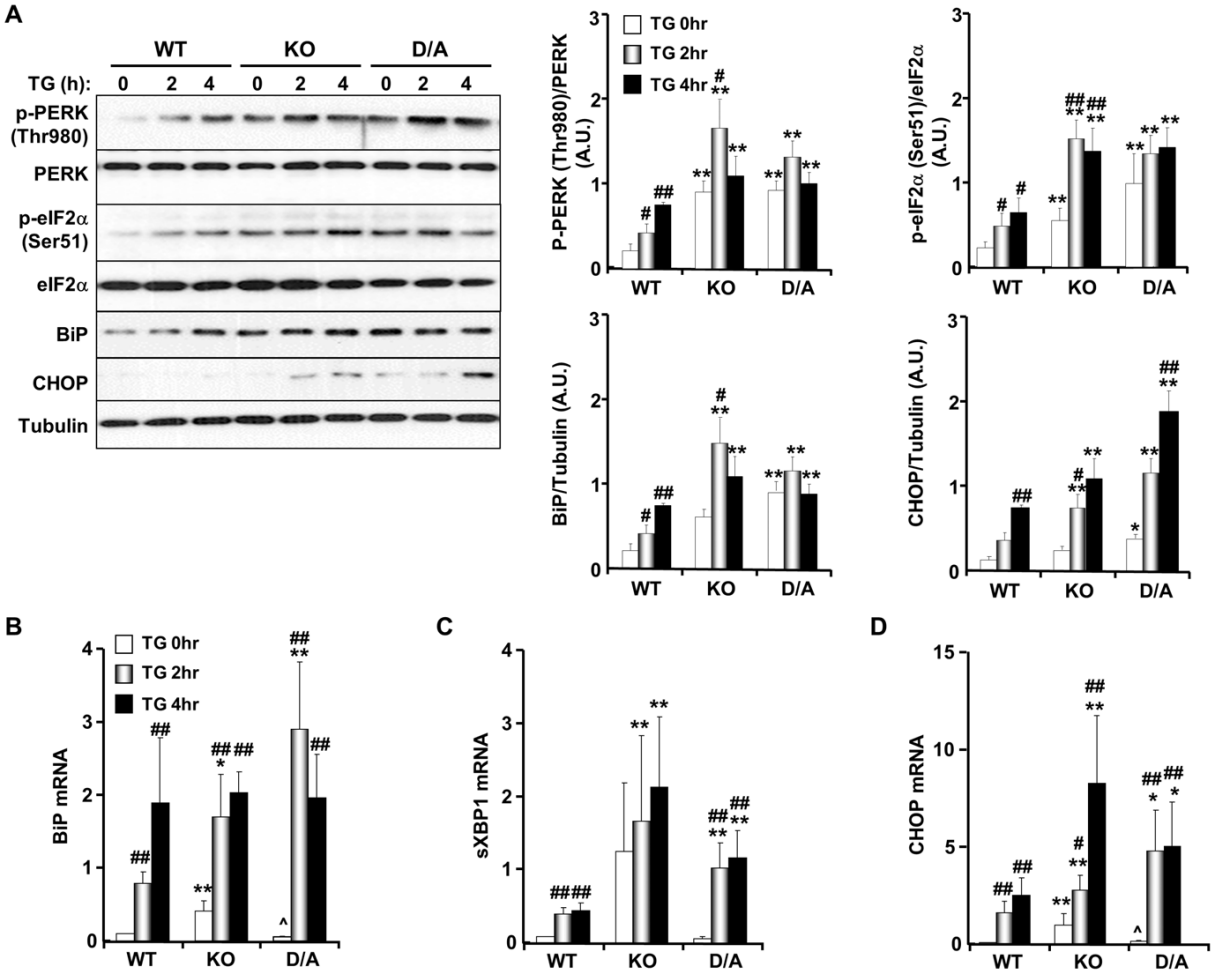
Enhanced PERK/eIF2α phosphorylation in PTP1B-deficient brown adipocytes. Differentiated brown adipocytes were treated with thapsigargin (TG; 1 µM) for the indicated times. (**A**) Immunoblots of p-PERK (Thr980), PERK, p-eIF2α (Ser51), eIF2α, BiP and CHOP in lysates of differentiated WT, KO and D/A adipocytes. BiP (**B**), sXBP1 (**C**) and CHOP (**D**) mRNA was measured by quantitative real-time PCR and normalized against GAPDH. Data represent means ± SEM of three independent experiments. (*) indicates significant difference between KO and D/A versus WT at the corresponding time, (^#^) indicates significant difference between treated and non-treated cells within each group, and (∧) indicates significant difference between KO and D/A at the corresponding time.

To determine whether the effects of PTP1B deficiency in BAT on UPR were cell autonomous, we utilized previously generated brown preadipocyte cell lines [42], [43]. Briefly, immortalized brown preadipocytes were generated from whole-body PTP1B KO mice and reconstituted with human (h) PTP1B (WT). It is noteworthy that hPTP1B shares a high degree of homology to mouse (m) PTP1B and functionally complements the loss of mPTP1B in mouse embryonic fibroblasts [44]. In addition, KO cells were reconstituted with substrate-trapping hPTP1B D181A (D/A) mutant that retains substrate binding but is catalytically impaired [42], [43]; therefore the mutant forms stable complexes with tyrosine-phosphorylated substrates. Differentiation of KO and reconstituted (WT and D/A) preadipocytes was performed as previously described [42], [43]. ER stress was induced in differentiated adipocytes by treatment with thapsigargin (TG) or tunicamycin (TN) [45], [46]. Treatment with TG resulted in higher PERK Thr980 and eIF2α Ser51 phosphorylation, BiP and CHOP expression in KO and D/A adipocytes compared with WT (Fig. 2A). In addition, increased BiP, sXBP1 and CHOP mRNA in response to ER stress was more evident in KO and D/A adipocytes compared with WT (Fig. 2B–D). Comparable findings were observed in TN-treated adipocytes indicating that the effects are not limited to TG-induced ER stress (Fig. S3). Together, these data demonstrate enhanced UPR in BAT and adipocytes lacking PTP1B.

### Adipose PTP1B deficiency increased PERK tyrosine phosphorylation

**Figure 3:**
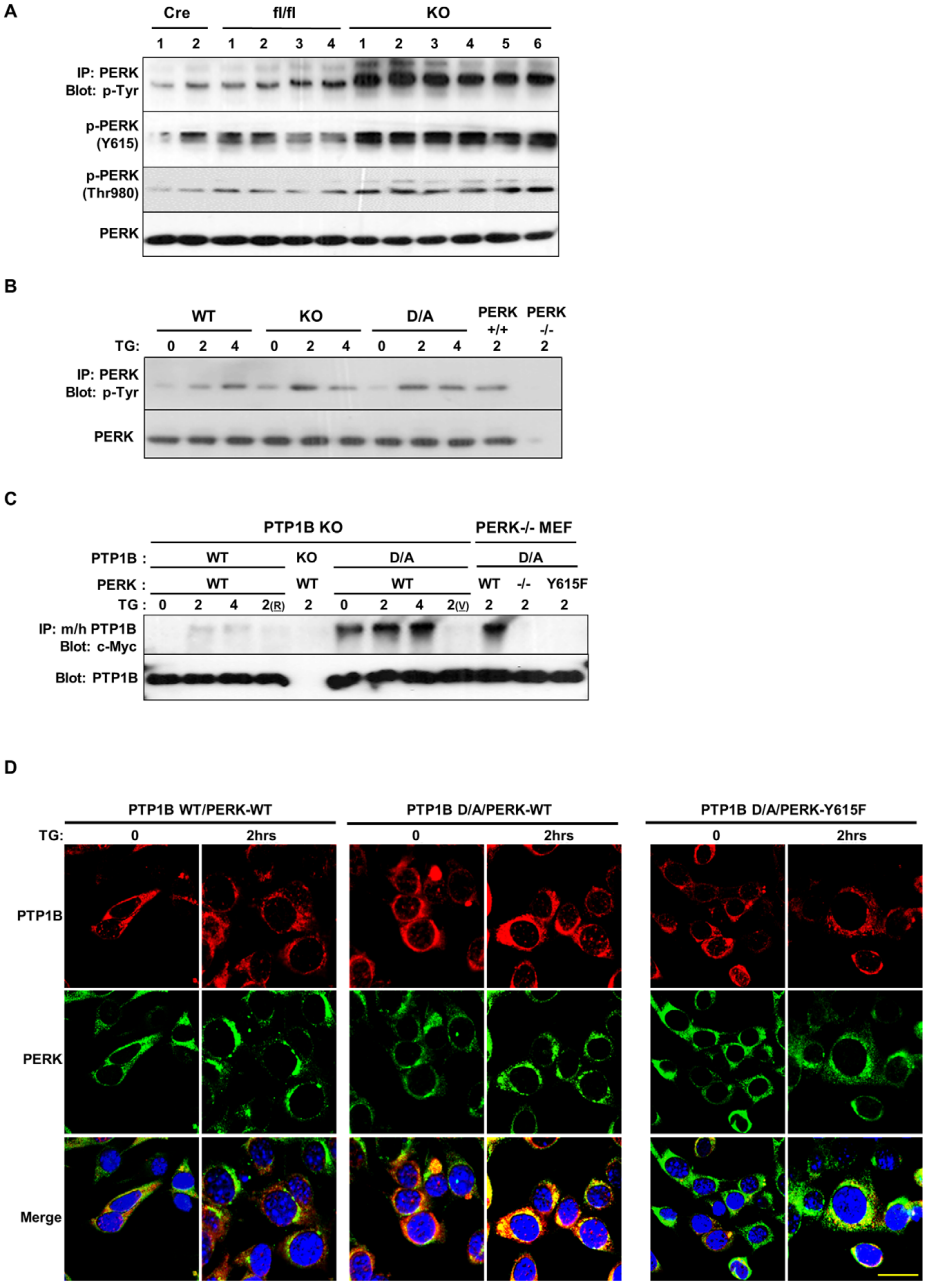
PTP1B dephosphorylation of PERK. (**A**) PERK was immunoprecipitated from BAT lysates of Cre, fl/fl and adipose-PTP1B KO (KO) male mice fed HFD for 30 weeks, and then immunoblotted with anti-phosphotyrosine antibodies, phospho-specific antibodies for PKR Tyr293 (PERK Y615) and PERK (Thr980). Blots were also probed for PERK to control for loading. Each lane represents brown adipose tissue from a different animal. (**B**) Differentiated WT, KO and D/A brown adipocytes and PERK−/− and PERK+/+ fibroblasts were treated with thapsigargin (TG) for the indicated times. Immunoprecipitates of PERK were immunoblotted with anti-phosphotyrosine antibodies. Blots were also probed for PERK to control for loading. (**C**) PTP1B KO preadipose cells were co-transfected with PTP1B WT and substrate-trapping D181A mutant (D/A) and Myc-tagged PERK wild type. In addition, PERK KO fibroblasts were co-transfected with PTP1B D/A and Myc-tagged PERK WT and Y615F mutant. Cell were treated with TG for the indicated times then lysed in NP40 or RIPA (R), with or without pervanadate (V) treatment. Lysates were immunoprecipitated using mouse (m) and human (h) PTP1B antibodies and immunoblotted using anti-c-Myc and anti-(m/h) PTP1B antibodies. (**D**) PTP1B KO brown preadipocytes were transfected with hPTP1B WT, or hPTP1B D/A (red) and c-Myc-PERK WT or Y615F mutant (green), treated with thapsigargin (TG) for 2 hours and visualized by fluorescence confocal microscopy. Scale bar corresponds to 20 µm.

Considering that tyrosine phosphorylation of PERK is required for optimal kinase activity [13], [14] and the function of PTP1B as a tyrosine phosphatase, we investigated whether PTP1B regulates PERK tyrosine phoshorylation. PERK was immunoprecipitated from BAT lysates of adipose-PTP1B KO and control mice and immunoblotted using anti-phosphotyrosine antibodies or phospho-specific antibodies that detect PERK Tyr615 and Thr980. PTP1B deficient BAT revealed increased overall PERK tyrosine phosphorylation compared with controls (Fig. 3A). In addition, PERK Tyr615 and Thr980 phosphorylation was enhanced in PTP1B deficient BAT compared with controls. In line with these observations, PERK overall tyrosine phosphorylation was increased in thapsigargin-treated KO and D/A adipocytes compared with WT (Fig. 3B). Togther, these data indicate inverse relationship between PTP1B activity and tyrosine phosphorylation of PERK in brown adipocytes.

These findings prompted us to examine whether PERK is a direct substrate of PTP1B. To that end, we transiently co-expressed wild type and substrate-trapping PTP1B (D/A) with wild type Myc-PERK in PTP1B KO preadipose cells and subjected to TG-induced ER stress. Cells were lysed as described in Methods and immunoprecipitates of PTP1B were probed for PERK using anti-Myc antibodies (Fig. 3C). No association between PERK and PTP1B WT was observed given the transient nature of PTP1B WT-substrate interactions. On the other hand, PERK co-immunoprecipitated only with PTP1B D/A at basal and TG-treated conditions, and co-association increased significantly after TG treatment. Notably, treatment with pervanadate (indicated with v in Fig. 3C), a strong inhibitor of tyrosine phosphatases, which oxidizes the essential cysteinyl residue in the catalytic center of the enzymes [47] disrupted PERK and PTP1B D/A interaction. This suggested that the association is consistent with enzyme-substrate interaction that is mediated by the active site cysteinyl residue. Given the increased phosphorylation of PERK Tyr615 in adipocytes lacking PTP1B, we tested the importance of this residue in mediating association with PTP1B D/A. PTP1B D/A was co-expressed with Myc-PERK WT and Myc-PERK Y615F mutant in PERK−/− fibroblasts, and subjected to TG-induced ER stress. Wild type PERK co-immunoprecipitated with PTP1B D/A as expected but mutation of Tyr615 abrogated PERK association with PTP1B D/A (Fig. 3C).

To evaluate the cellular dynamics of PTP1B and PERK in response to ER stress, we transiently co-expressed Cherry-PTP1B WT or Cherry-PTP1B D/A with Myc-PERK (WT or Y615F) in brown preadipocytes and monitored their sub-cellular localization under basal and TG-induced ER stress using confocal microscopy (Fig. 3D). Consistent with previous reports [22], [23], [48], PTP1B and PERK localized to the ER network. In addition, wild type PERK co-localized with PTP1B D/A under basal and TG-treated conditions. Notably, mutating Tyr615 in PERK significantly attenuated co-localization with PTP1B D/A in line with biochemical data. Collectively, these data established PERK as a direct substrate for PTP1B in adipocytes and revealed that the interaction is mediated through PERK Tyr615.

### PTP1B deficiency increased ATF4 translation in brown adipocytes

**Figure 4:**
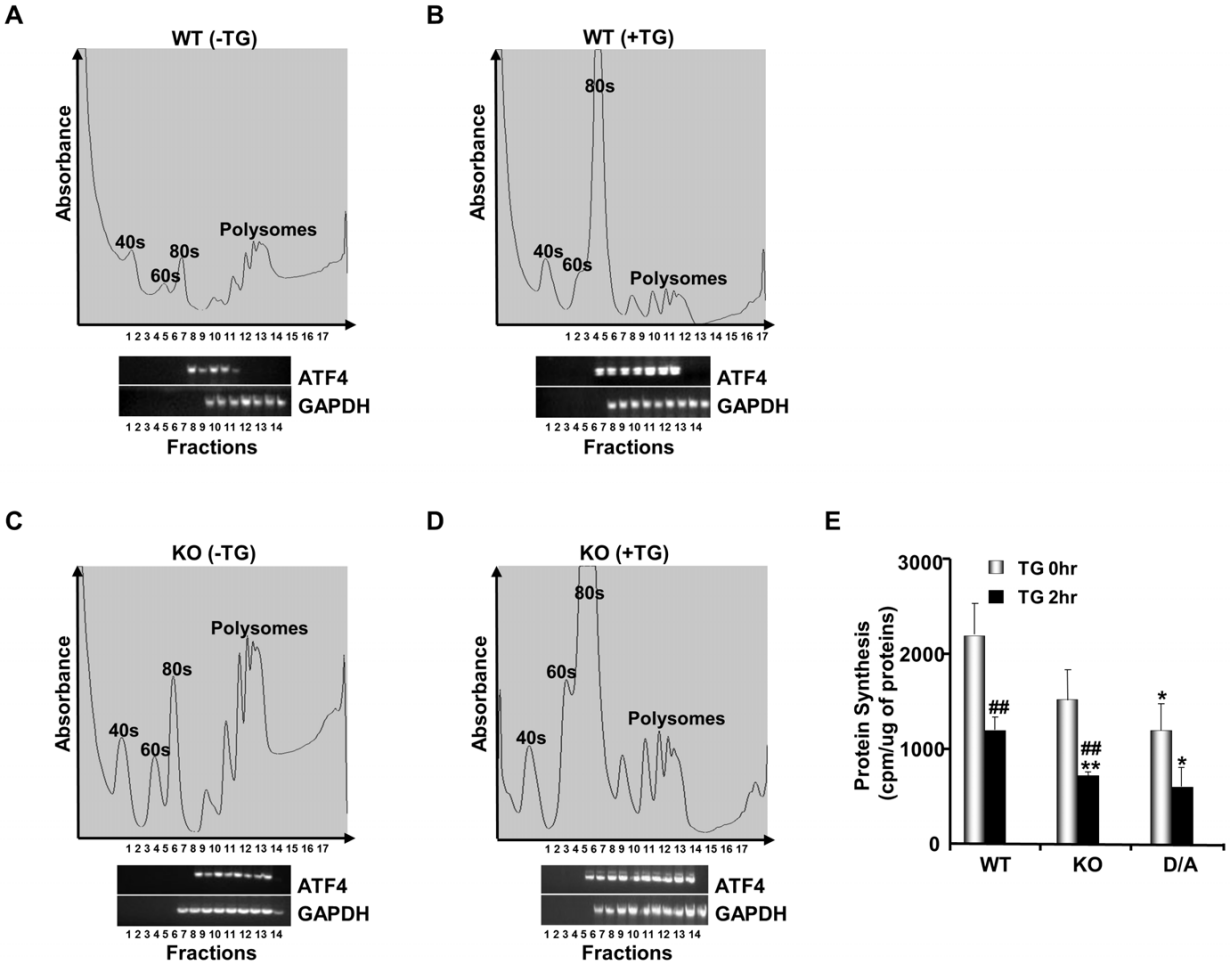
Enhanced ATF4 mRNA translation in PTP1B-deficient differentiated brown adipocytes. Differentiated WT (**A, B**) and PTP1B KO (**C, D**) brown adipocytes were left untreated (**A, C**) or treated (**B, D**) with thapsigargin (TG, 1 µM) for 2 hr followed by polysome profile analysis using 20–50% sucrose gradients as described in Methods. Gradients were fractionated from top (fraction 1) to bottom (fraction 17). Absorbance of the gradients was measured continuously at 264 nm to provide the polysome profiles. Association of ATF4 mRNA and GAPDH mRNA with the ribosomal interface was detected by RT-PCR. Data present one of two reproducible experiments. **E**) Protein synthesis in differentiated WT, KO and D/A brown adipocytes before and after TG treatment. Bar chart represents newly incorporated ^35^S methionine from three independent experiments. (*) indicates significant difference between KO and D/A versus WT at the corresponding treatment time and (^#^) indicates significant difference between treated and non-treated cells within each group.

eIF2α Ser51 phosphorylation plays a critical role in blocking translational initiation [49]. Paradoxially, eIF2α phosphorylation increases translation of ATF4 mRNA to produce a transcription factor that activates expression of several UPR target genes [50]. The observed increase in eIF2α phosphorylation in BAT and adipocytes lacking PTP1B prompted us to examine the translational effects of PTP1B on endogenous ATF4 mRNA in response to ER stress. Differentiated KO and WT brown adipocytes were subjected to polysome profile analysis, a technique that allows the separation of monosomes from polyribosomes by sucrose density centrifugation [51] (Fig. 4). Efficiently translated mRNAs are bound to polyribosomes (polysomes) whereas poorly translated mRNAs are detected in fractions with monosomes. Under basal conditions, we observed that a larger amount of ATF4 mRNAs was associated with polyribosomes in PTP1B KO adipocytes compared with WT (Fig. 4A, C). Upon treatment with TG, we observed that the ATF4 transcripts shifted towards larger polyribosomes in both cell types (Fig. 4B, D) indicating their efficient translation as a result of PERK activation and eIF2α phosphorylation. Interestingly, however, a significant fraction of ATF4 mRNA shifted to heavier polysomes in TG-treated KO cells compared with WT cells. This difference in ATF4 mRNA translation is consistent with the higher eIF2α phosphorylation in TG-treated KO adipocytes than in WT.

To determine protein synthesis in WT, KO and D/A adipocytes, we used ^35^S-methionine metabolic labelling as described in Methods. Under basal condition, KO and D/A adipocytes exhibited lower protein synthesis compared with WT (Fig. 4E). TG treatment drastically diminished protein synthesis by 45% in WT cells, and resulted in greater reduction (60 and 50%) in KO and D/A cells, respectively. These findings are consistent with eIF2α Ser51 phosphorylation and polysome profile analyses in these cells. Together, our data indicate that the control of PERK-eIF2α phosphorylation by PTP1B in brown adipocytes signals to translational machinery through the regulation of the translatability of ATF4 mRNA in the absence as well as presence of ER stress.

## Discussion

PTP1B is a physiological regulator of systemic glucose homeostasis and energy balance. Given the salutary effects of whole-body PTP1B deficiency, it is an attractive target for therapy of diabetes and/or obesity. Obesity leads to increased ER stress in insulin-responsive tissues [3], [6], but the precise contribution of ER stress to metabolic regulation, and modulation of ER stress by PTP1B requires additional investigation. In this study, we identified PTP1B as a key regulator of PERK/eIF2α sub-arm of ER stress signaling in brown adipocytes. We demonstrated enhanced phosphorylation and activation of PERK and eIF2α in BAT of mice lacking PTP1B challenged with HFD and in differentiated brown adipocytes treated with thapsigargin and tunicamycin. At the molecular level, we uncovered direct interaction between PERK and PTP1B in adipocytes. PERK is modified by tyrosine phosphorylation and its tyrosine phosphorylation is highly induced in response to ER stress [14]. Indeed, our biochemical studies revealed significant co-association between PERK and the substrate-trapping mutant PTP1B D/A at basal conditions. Increased tyrosine phosphorylation of PERK after TG treatment resulted in concomitant increase in PERK-PTP1B D/A co-association. Of note, PTP1B-PERK association is consistent with enzyme-substrate interaction that is mediated by the active site cysteinyl residue of PTP1B since pervanadate treatment disrupts the interaction. In addition, we identified PERK Tyr615 as a key mediator of interaction with PTP1B. Notably, PERK Tyr615 phosphorylation is required for maximal kinase activity *in vitro* and *in vivo* [14]. In line with increased PERK phosphorylation in adipocytes lacking PTP1B, eIF2α Ser51 phosphorylation was elevated resulting in better translatability of ATF4 mRNA.

Prior reports indicated that deletion of PTP1B in liver can reduce activation of PERK (Thr980 phosphorylation) and eIF2α phosphorylation in response to certain stresses such as high fat diet, suggesting that PTP1B potentially promotes activation of PERK [28], [29]. Similarly, mouse embryonic fibroblasts lacking PTP1B exhibit impaired IRE1α signaling and attenuated ER stress-induced apoptosis [27]. By contrast, our current studies show clearly that PTP1B deficiency enhanced PERK/eIF2α sub-arm of ER stress response in BAT and adipocytes. These findings are in line with previous studies in MIN6 insulinoma β-cells where PTP1B deficiency enhances palmitate- or tunicamycin-induced PERK/eIF2α ER stress signaling, whereas PTP1B overexpression suppresses this ER stress response [30]. Related to these findings, recent elegant studies demonstrate that PTP1B is inhibited by sulfhydration in response to endogenously generated hydrogen sulfide [52]. Importantly, PTP1B inhibition directly promotes PERK activity (and Tyr615 phsophorylation) during the response to ER stress [52]. The reason(s) for discrepancies between the studies is currently not clear. Conceivably, PTP1B has distinct substrates and/or regulates distinct arms of ER stress signaling in different tissues and in response to various challenges. Alternatively, PTP1B may affect the same pathways in different tissues, but the effects of those pathways may differ in a tissue-specific manner. Indeed, induction of ER stress in adipocytes and liver cells using protease inhibitors leads to downregulation of lipogenic genes in adipocytes and upregulation in hepatocytes [53]. Finally, it is worth noting that the effects of PTP1B on ER stress response could be general; caused by indirect regulation of one or more sub-arm of ER stress pathway, and/or specific; caused by direct regulation of PERK signaling.

In summary, we identified PTP1B as a key regulator of PERK/eIF2α signaling and established PERK as a direct substrate of PTP1B in adipocytes. Importantly, we determined functional cross talk between PTP1B and PERK, and demonstrated that PTP1B deficiency in adipocytes regulates the translational machinery by directly modulating PERK signaling. Of note, we cannot rule out that PTP1B-PERK interaction leads to regulation of PTP1B activity by PERK. This may be implied by findings that PKR can stimulate the activity of the closely-related T cell PTP [54]. At any rate, our findings provided new insights into the regulation of adipocyte ER stress signaling by PTP1B. Additional studies will provide further insights into the complex regulation of ER stress by PTP1B in various tissues and in response to different challenges, and decipher its contribution to metabolic regulation.

## Materials and Methods

### Mouse studies

PTP1B-floxed (PTP1B^fl/fl^) mice were generated previously [55]. Adiponectin (Adipoq)-Cre mice were obtained from Dr. E. Rosen (BIDMC/Harvard University). Mice were maintained on a 12-hour light-dark cycle in a temperature-controlled facility, with free access to water and food. Mice were fed high fat diet (HFD; 60% Kcal from fat, Cat # D12492, Research Diets) at weaning. Genotyping for the PTP1B floxed allele and presence of Cre was performed by polymerase chain reaction (PCR), using DNA extracted from tails [55]. All mouse studies were approved by the Institutional Animal Care and Use Committee at the University of California Davis.

### Cell culture

Primary brown preadipocytes were isolated from wild type and whole-body PTP1B KO mice and immortalized as previously described [42], [56], [57]. PTP1B KO cells were retrovirally reconstituted with wild type human (h) PTP1B (WT) or substrate-trapping mutant D181A (D/A) as we previously described [44]. Cells were induced to differentiate into brown adipocytes with massive accumulation of multilocular fat droplets using established protocols [56], [58]. ER stress was induced in differentiated adipocytes by treatment with tunicamycin (2 ng/ml) or thapsigargin (1 µM). PERK−/− and PERK+/+ fibroblasts were cultured as previously described [59].

### Biochemical analyses

Brown adipose tissues were ground in the presence of liquid nitrogen and lysed using radio-immunoprecipitation assay (RIPA) buffer (10 mM Tris-HCl, pH 7.4, 150 mM NaCl, 0.1% sodium dodecyl sulfate [SDS], 1% Triton X-100, 1% sodium deoxycholate, 5 mM EDTA, 1 mM NaF, 1 mM sodium orthovanadate and protease inhibitors). Lysates were clarified by centrifugation at 13,000 rpm for 10 min, and protein concentrations were determined using a bicinchoninic acid protein assay kit (Pierce Chemical). For differentiated brown adipocytes, cells were lysed in RIPA and extracts were sonicated then clarified as indicated before. For substrate-trapping experiments, cells were lysed in 1% NP40 buffer with a protease inhibitor cocktail (without sodium pervanadate). Immune complexes were collected on protein G-Sepharose beads (GE Healthcare) and washed with lysis buffer. Proteins were resolved by SDS-PAGE and transferred to PVDF membranes. Immunoblots were performed with phospho-tyrosine antibodies 4G10 (Upstate Biotechnology) and PY99, or antibodies against p-PERK (Thr980), PERK, p-eIF2α (Ser51), eIF2α, sXBP1, ATF6α, BiP and CHOP (all from Santa Cruz Biotechnology). Anti-p-PERK (Tyr615) antibodies were previously described [14]. Proteins were visualized using enhanced chemiluminescence (ECL, Amersham Biosciences) and pixel intensities of immuno-reactive bands were quantified using FluorChem 9900 (Alpha Innotech, CA).

Total RNA was extracted from BAT and differentiated adipocytes using TRIzol reagent (Invitrogen). cDNA was generated using high-capacity cDNA Archive Kit (SuperScript™ III Reverse Transcriptase, Invitrogen). mRNA of BiP, spliced (s)XBP1, CHOP, and ATF4 was assessed by quantitative reverse transcription PCR (iCycler, BioRad) with appropriate primers (Table S1) and normalized to glyceraldehyde 3-phosphate dehydrogenase (GAPDH).

### Polysome profile analyses

Polysome profiles and detection of mRNAs by RTPCR were performed as previously described [51], [60]. Briefly, cytoplasmic extracts of differentiated cells with and without thapsigargin treatment were carefully layered over 10 to 50% linear sucrose gradients in polysome buffer (300 mM NaCl, 150 mM Tris, pH 7.5, 5 mM MgCl_2_, 1 mM dithiothreitol, 50 U of recombinant RNasin, and 0.5% sodium deoxycholate) and centrifuged at 40,000 rpm in a Beckman SW40Ti rotor for 2.5 h at 4°C. Gradients were fractionated using an ISCO gradient fractionation system equipped with a UA-6 detector following an upward displacement method. Light RNP fractions, 40S, 60S, 80S, and heavy polysome fractions were monitored by continuous UV absorption profiles at absorbance of 264 nm and 800 µl fractions were collected. mRNAs from the translationally active (polysome) and silenced pools (mRNP/monosome) were isolated from the fractions and ATF4 expression determined using RT-PCR.

### [^35^S] metabolic pulse labeling

Protein synthesis was measured as previously described [61]. Briefly, differentiated adipocytes were starved overnight in DMEM lacking methionine and supplemented with 10% dialyzed fetal bovine serum. Tran^35^S-label (ICN) was then added to the cells at concentration of 100 µCi/10^6^ cells for 30 min followed by thapsigargin treatment for 2 hrs. Cells were washed with PBS then lysed in RIPA buffer. 250 µg of total protein were precipitated using trichloroacetic acid at concentration of 10% w/v, washed with 300 µl of acetone, resuspended in 100 µl of PBS-2% SDS and transferred to 4.9 ml of scintillation fluid. The incorporated radioactivity was quantitated using liquid scintillation counting.

### Statistical analyses

Data are expressed as means ± standard error of the mean (SEM). All statistical analyses were performed using JMP Statistical Discovery (SAS Institute). For experiments on mouse tissues, statistical analyses were performed using one-way ANOVA and Bonferroni-Holmes method for Post hoc analysis. For experiments on cells, statistical analyses were performed using Student's *t* test. A symbol (such as *) indicates P≤0.05, whereas a duplicate symbol (such as **) indicates P≤0.01.

## Supporting Information

Figure S1 **ER stress induction leads to increased PTP1B expression in adipose tissue and adipocytes.** **A**) Immunoblots of PTP1B in brown (upper panel) and subcutaneous (lower panel) adipose tissue depots from mice fed chow and HFD for 3 or 11 weeks. Each lane represents adipose tissue from a different animal. **B**) PTP1B expression in differentiated brown (upper panel) and white (lower panel) adipocytes upon exposure to 0.5 mM palmitate for the indicated times. Blots were reprobed for Tubulin to control for loading. Bar charts represent PTP1B expression normalized to Tubulin. In panel A (*) indicates significant difference between chow and HFD fed mice in BAT and SQ depot. In panel B (*) indicates significant difference between palmitate-treated and non-treated cells.
(TIF)

Figure S2 **Enhanced UPR in PTP1B-deficient WAT depots.** Immunoblots of p-PERK (Thr980), PERK, p-eIF2α (Ser51), eIF2α, BiP, sXBP1, cATF6α and CHOP in subcutaneous (**A**) and retroperitoneal (**B**) adipose depots from fl/fl and adipose-PTP1B KO mice fed HFD for 26 weeks. Lysates were also probed for Tubulin to control for loading. Each lane represents tissue from a different animal. Bar charts represent p-PERK (Thr980) and p-eIF2α (Ser51) normalized to their protein expression, BiP, sXBP1, cATF6α and CHOP normalized to Tubulin. Data represent means ± SEM from at least five different mice per genotype. (*) indicates significant difference between KO and fl/fl mice.
(TIF)

Figure S3 **Enhanced PERK/eIF2α phosphorylation in PTP1B-deficient brown adipocytes exposed to tunicamycin.** Differentiated brown adipocytes were treated with tunicamycin (TN; 2 ng/ml) for 12 or 24 hours. (**A**) Immunoblots of p-PERK (Thr980), PERK, p-eIF2α (Ser51), eIF2α, BiP and CHOP in lysates of differentiated WT, KO and D/A adipocytes. BiP (**B**), sXBP1 (**C**) and CHOP (**D**) mRNA was measured by quantitative real-time PCR and normalized against GAPDH. Data represent means ± SEM of three independent experiments. (*) indicates significant difference between KO and D/A versus WT at the corresponding time, (^#^) indicates significant difference between treated and non-treated cells within each group, and (∧) indicates significant difference between KO and D/A at the corresponding time.
(TIF)

Table S1 **Primers sequences used for real time PCR.**
(TIF)
