# Supplementary figures and images for: Protein Tyrosine Phosphatase 1B Deficiency Potentiates PERK/eIF2α Signaling in Brown Adipocytes
Source: PLoS One. 2012 Apr 3;7(4):e34412. doi: 10.1371/journal.pone.0034412 (PMC3317973; doi:10.1371/journal.pone.0034412)

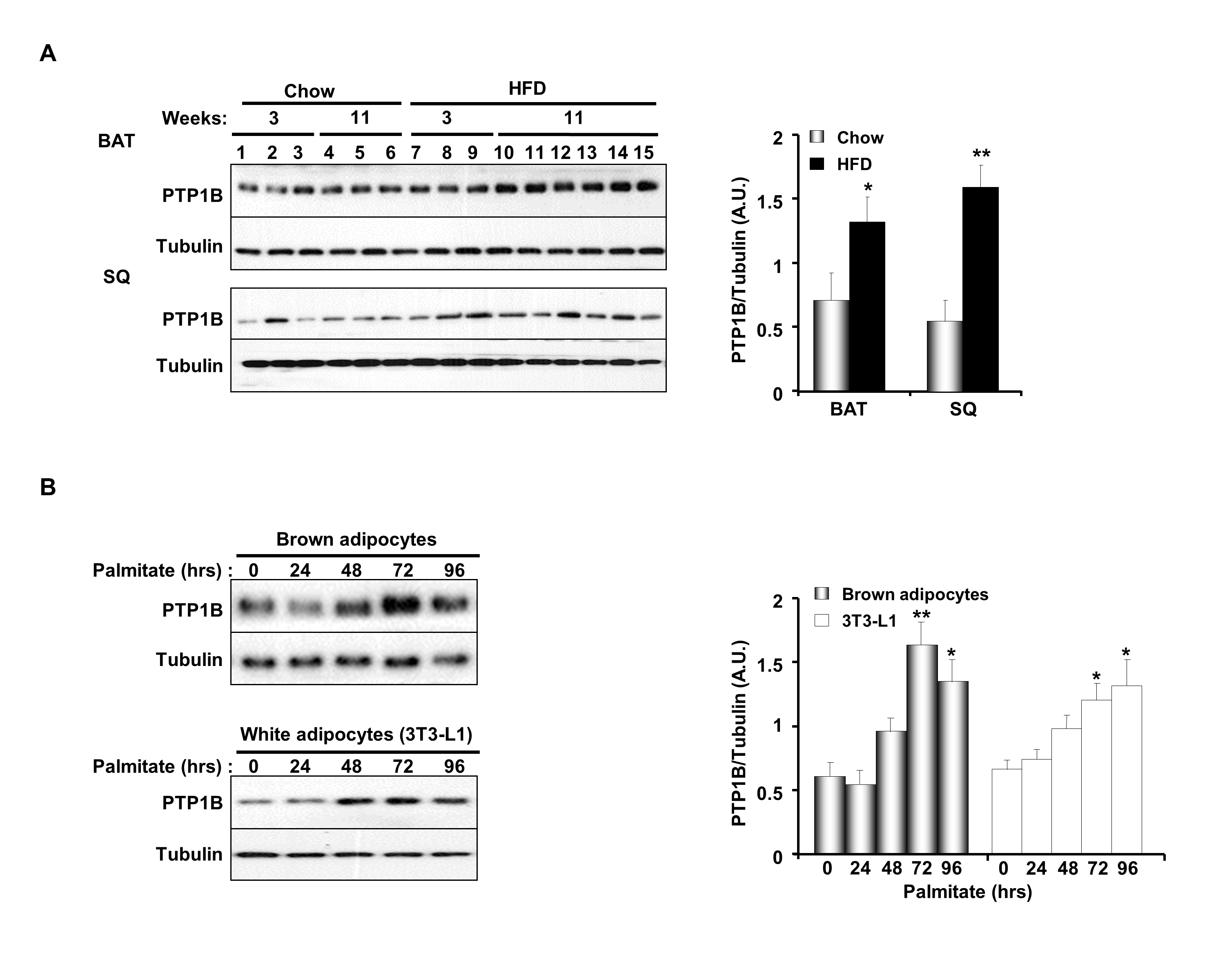

Supplement: Figure S1 — ER stress induction leads to increased PTP1B expression in adipose tissue and adipocytes. A) Immunoblots of PTP1B in brown (upper panel) and subcutaneous (lower panel) adipose tissue depots from mice fed chow and HFD for 3 or 11 weeks. Each lane represents adipose tissue from a different animal. B) PTP1B expression in differentiated brown (upper panel) and white (lower panel) adipocytes upon exposure to 0.5 mM palmitate for the indicated times. Blots were reprobed for Tubulin to control for loading. Bar charts represent PTP1B expression normalized to Tubulin. In panel A (*) indicates significant difference between chow and HFD fed mice in BAT and SQ depot. In panel B (*) indicates significant difference between palmitate-treated and non-treated cells. (TIF) [file pone.0034412.s001.tif]

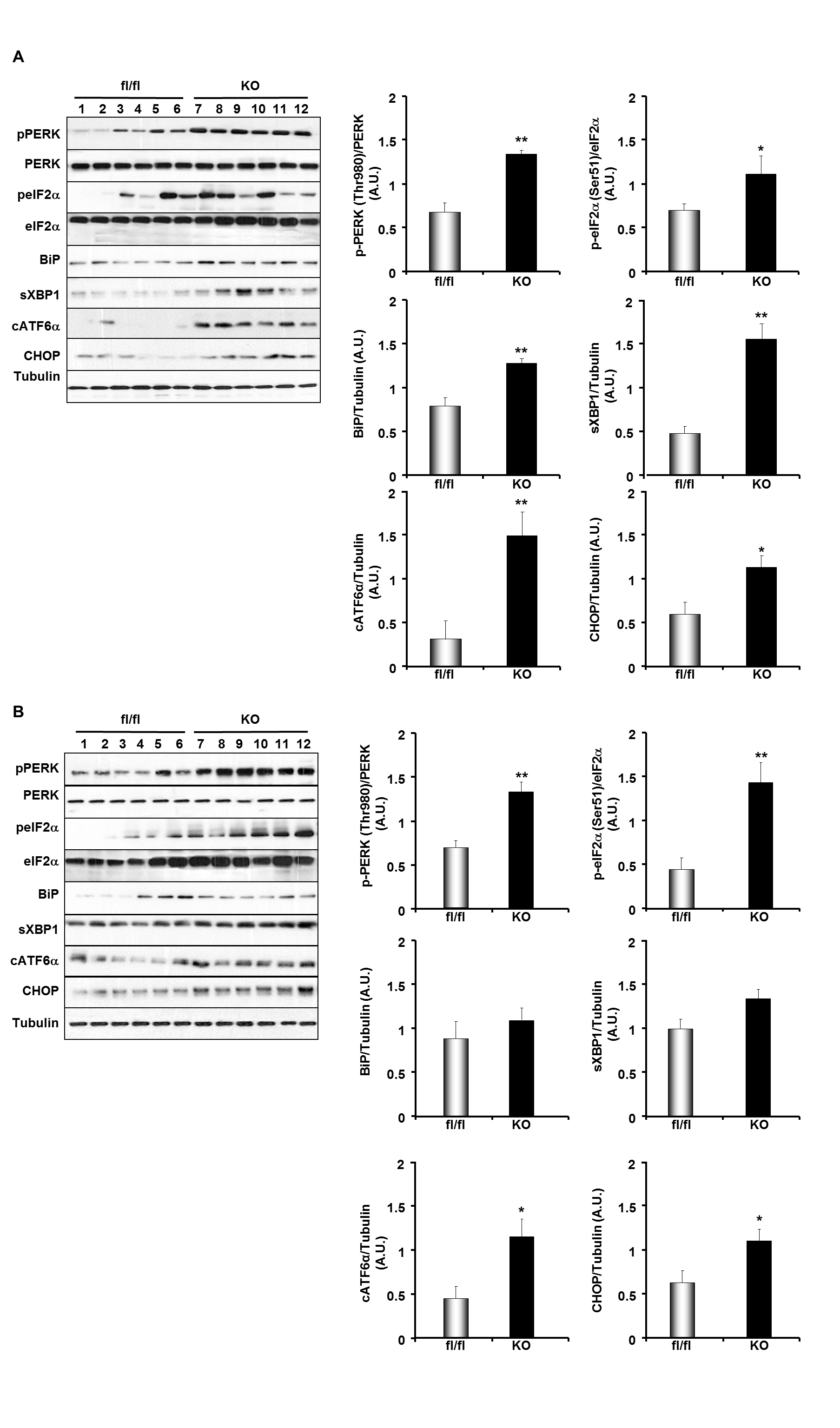

Supplement: Figure S2 — Enhanced UPR in PTP1B-deficient WAT depots. Immunoblots of p-PERK (Thr980), PERK, p-eIF2α (Ser51), eIF2α, BiP, sXBP1, cATF6α and CHOP in subcutaneous (A) and retroperitoneal (B) adipose depots from fl/fl and adipose-PTP1B KO mice fed HFD for 26 weeks. Lysates were also probed for Tubulin to control for loading. Each lane represents tissue from a different animal. Bar charts represent p-PERK (Thr980) and p-eIF2α (Ser51) normalized to their protein expression, BiP, sXBP1, cATF6α and CHOP normalized to Tubulin. Data represent means ± SEM from at least five different mice per genotype. (*) indicates significant difference between KO and fl/fl mice. (TIF) [file pone.0034412.s002.tif]

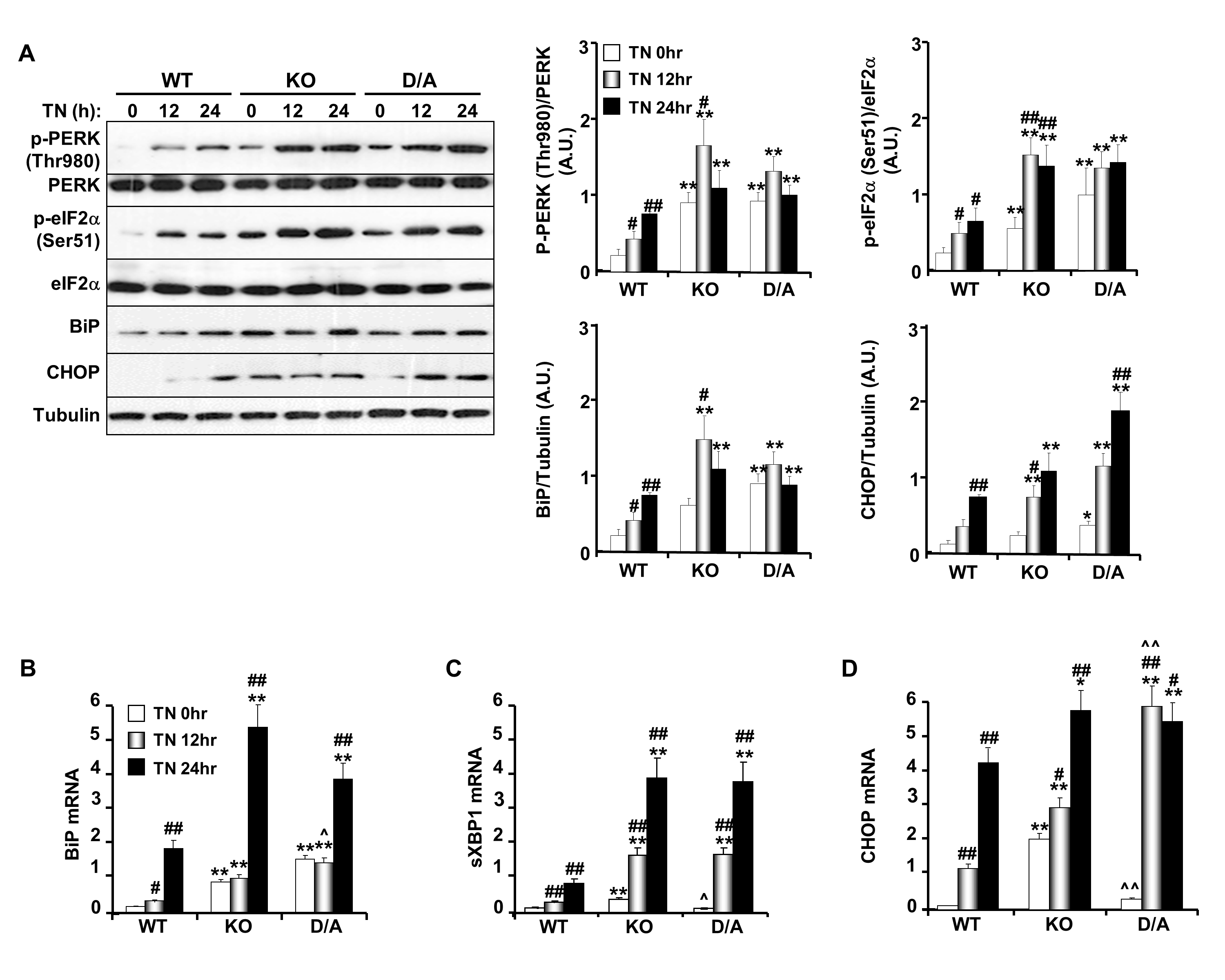

Supplement: Figure S3 — Enhanced PERK/eIF2α phosphorylation in PTP1B-deficient brown adipocytes exposed to tunicamycin. Differentiated brown adipocytes were treated with tunicamycin (TN; 2 ng/ml) for 12 or 24 hours. (A) Immunoblots of p-PERK (Thr980), PERK, p-eIF2α (Ser51), eIF2α, BiP and CHOP in lysates of differentiated WT, KO and D/A adipocytes. BiP (B), sXBP1 (C) and CHOP (D) mRNA was measured by quantitative real-time PCR and normalized against GAPDH. Data represent means ± SEM of three independent experiments. (*) indicates significant difference between KO and D/A versus WT at the corresponding time, (#) indicates significant difference between treated and non-treated cells within each group, and (∧) indicates significant difference between KO and D/A at the corresponding time. (TIF) [file pone.0034412.s003.tif]

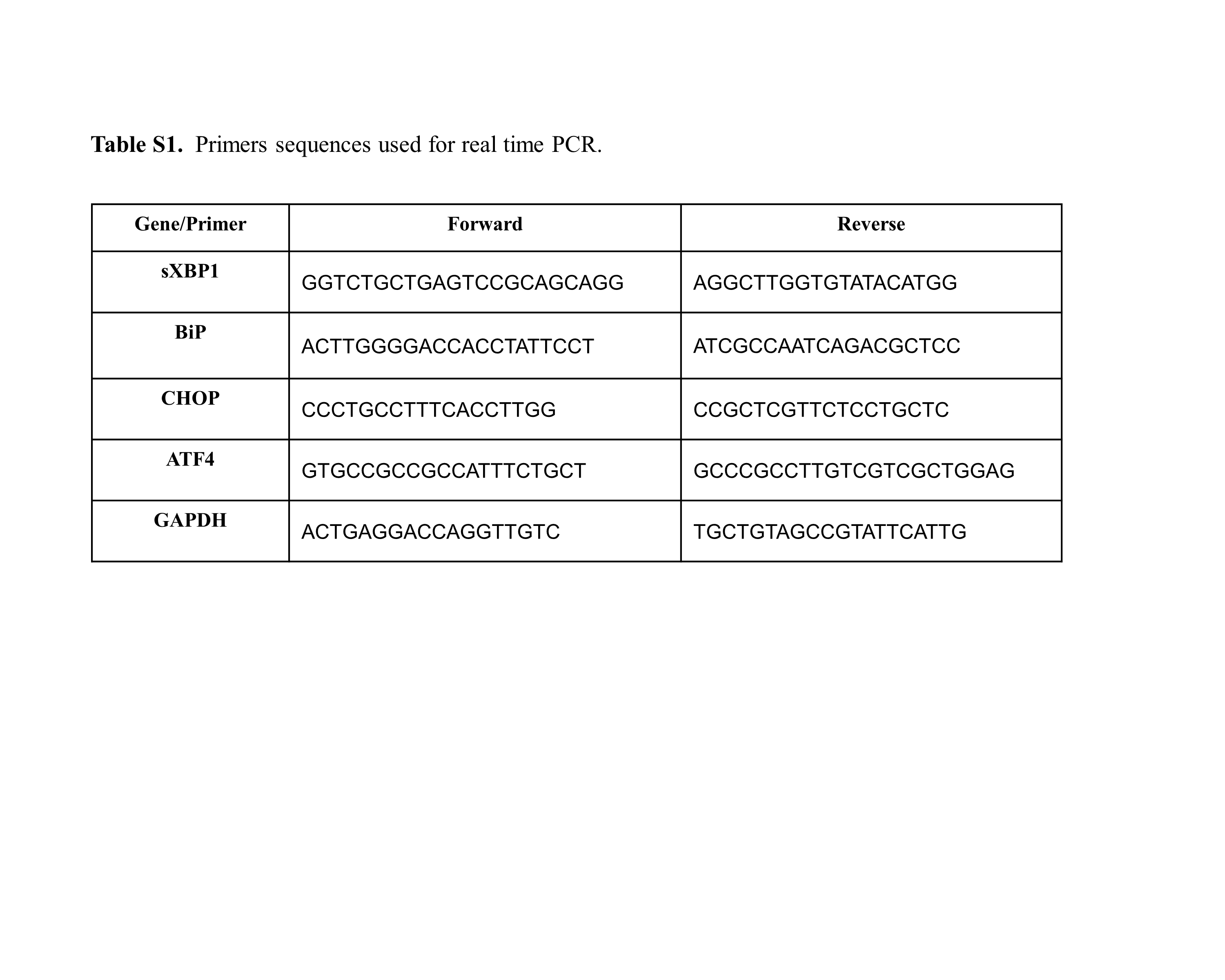

Supplement: Table S1 — Primers sequences used for real time PCR. (TIF) [file pone.0034412.s004.tif]
